# Modular Evolution of DNA-Binding Preference of a Tbrain Transcription Factor Provides a Mechanism for Modifying Gene Regulatory Networks

**DOI:** 10.1093/molbev/msu213

**Published:** 2014-07-12

**Authors:** Alys M. Cheatle Jarvela, Lisa Brubaker, Anastasia Vedenko, Anisha Gupta, Bruce A. Armitage, Martha L. Bulyk, Veronica F. Hinman

**Affiliations:** ^1^Department of Biological Sciences, Carnegie Mellon University; ^2^Division of Genetics, Department of Medicine, Brigham and Women’s Hospital and Harvard Medical School, Boston, MA; ^3^Department of Chemistry, Carnegie Mellon University; ^4^Department of Pathology, Brigham and Women’s Hospital and Harvard Medical School, Boston, MA

**Keywords:** evo-devo, gene regulatory networks, transcription factors, echinoderm, evolution, T-box

## Abstract

Gene regulatory networks (GRNs) describe the progression of transcriptional states that take a single-celled zygote to a multicellular organism. It is well documented that GRNs can evolve extensively through mutations to *cis*-regulatory modules (CRMs). Transcription factor proteins that bind these CRMs may also evolve to produce novelty. Coding changes are considered to be rarer, however, because transcription factors are multifunctional and hence are more constrained to evolve in ways that will not produce widespread detrimental effects. Recent technological advances have unearthed a surprising variation in DNA-binding abilities, such that individual transcription factors may recognize both a preferred primary motif and an additional secondary motif. This provides a source of modularity in function. Here, we demonstrate that orthologous transcription factors can also evolve a changed preference for a secondary binding motif, thereby offering an unexplored mechanism for GRN evolution. Using protein-binding microarray, surface plasmon resonance, and in vivo reporter assays, we demonstrate an important difference in DNA-binding preference between Tbrain protein orthologs in two species of echinoderms, the sea star, *Patiria miniata*, and the sea urchin, *Strongylocentrotus purpuratus*. Although both orthologs recognize the same primary motif, only the sea star Tbr also has a secondary binding motif. Our in vivo assays demonstrate that this difference may allow for greater evolutionary change in timing of regulatory control. This uncovers a layer of transcription factor binding divergence that could exist for many pairs of orthologs. We hypothesize that this divergence provides modularity that allows orthologous transcription factors to evolve novel roles in GRNs through modification of binding to secondary sites.

## Introduction

Animal morphology arises under the control of interacting networks of regulatory genes that operate during embryonic development. A central pursuit for understanding evolution of animal form is therefore to determine how these gene regulatory networks (GRNs) evolve. Several influential articles, published almost 50 years ago, set forth the hypothesis that noncoding DNA, that is, the *cis* regulatory DNA, would be the predominant source of evolutionary change. This idea was first predicted by Monod and Jacob (1961) who emphasized the important distinction between biochemical protein function and context of the action of that protein. Britten and Davidson (1971) established the hypothesis that regulatory mutations, which control this context, would be the prominent source of evolutionary variation. In 1975, King and Wilson suggested that the stark differences in morphology and behavior between chimpanzees and humans, despite their overall high similarity in DNA sequence, could be the result of differences in their regulatory DNA. These, and other articles of this era, firmly established the notion that changes to the deployment of genes, rather than the biochemical function of genes would be the main driver in morphological diversity. The rationale for this is theoretically straightforward. A single gene is usually regulated by multiple *cis*-regulatory modules (CRMs; and also referred to as enhancers), so that its expression in distinct spatial and temporal domains is governed independently. By comparison, the transcription factors that utilize these CRMs must remain evolutionarily dormant because they often are needed to orchestrate a variety of crucial tasks. This tends to be especially evident during development where transcriptions factors are used in multiple contexts. It stands to reason that mutations to CRMs have fewer pleiotropic effects and are therefore more likely to pass the filter of selection and thus these become the source of novelty and change (reviewed in Carroll 2005; Prud’homme et al. 2007; Wray 2007).

Many early discoveries in evolutionary developmental biology supported this hypothesis. A wealth of data demonstrates that all animals share highly similar sets of regulatory genes, which have been dubbed the toolkit for development (Carroll 2005). Regulatory genes comprise a relatively small portion of the transcriptome and hence must be used in many tissues and times in the developing embryo. Elegant xeno-transfer experiments further cemented the idea that regulatory proteins were evolutionarily dormant (McGinnis et al. 1990; Wang et al. 2002, 2004). One of the most exciting of these was the demonstration that the mouse *pax6* gene could rescue the mutant phenotypes of the eyes absent ortholog in *Drosophila* and had therefore presumably changed very little in the 900 My (Hedges et al. 2006) since insects and vertebrates last shared a common *pax6* gene (Halder et al. 1995).

More recently, a growing body of evidence suggests that although transcription factors may be a less common source of GRN evolutionary change, they are certainly not unchanging (Galant and Carroll 2002; Ronshaugen et al. 2002; Lynch and Wagner 2008; Chen et al. 2010; Nakagawa et al. 2013). In fact, the transcription factors that specify chemosensory neurons in *Caenorhabditis* acquired more nonsynonymous mutations than the chemosensory structural genes that they regulate in the same evolutionary distance (Jovelin 2009). Evolutionary changes occur in protein–protein interactions (Löhr and Pick 2005; Brayer et al. 2011) and posttranslational modifications (Lynch et al. 2011). The aforementioned examples explain how Ftz switched from a homeotic to a segmentation gene in insects and events contributing to the evolution of pregnancy as a novel feature in mammals, respectively. In very rare instances, evolutionary changes are also found within DNA consensus motif recognition (Hanes and Brent 1989; Baker et al. 2011). In the case of Bicoid, this new specificity is crucial for its function in directing anterior patterning in the *Drosophila* embryo (Hanes et al. 1994). Changes to DNA binding appear to be the rarest because unlike changes to the transcription factor’s cohort of protein-binding partners and posttranslational regulation, these presumably affect all instances of their function.

New technologies can determine DNA-binding motifs with greater sensitivities, particularly protein-binding microarrays (Berger et al. 2006). These arrays are designed with double-stranded DNA oligonucleotides of all possible *k*-mers, usually 44,000 oligonucleotides of 60 bp (with a 35 bp variable region). This provides 32-fold coverage of all possible 8-mer sequences. Protein binding to all oligonucleotides is measured, and position weight matrices that best represent binding sequence preferences are compiled. This type of data demonstrates that transcription factor-DNA interactions are more complex than originally imagined. In a survey of mouse transcription factor-binding preferences, nearly half of the proteins display binding preference for two distinct motifs; these have been termed their primary and secondary motifs (Badis et al. 2009). Secondary motifs are built when a single position weight matrix is unable to explain all of the highly bound sequences from the array data. Equally intriguing was the realization that these secondary motifs frequently differ for closely related paralogs. Presumably, this provides a mechanism through which paralogs may evolve. Upon duplication, one gene paralog can acquire new functions whereas the other maintains original functions. The in vivo functional significance of this additional component of binding specificity is still largely unknown, although a number of studies demonstrate that the binding motifs that do not match the primary consensus motif are not only present in endogenous CRMs but are often functionally distinct from the primary motif (Rowan et al. 2010; Parker et al. 2011; Busser et al. 2012; Zhu et al. 2012). Orthologs, which arise when species diverge instead of through gene duplication, experience greater evolutionary constraint, as they must maintain original functional roles while acquiring changes. Little is known about whether such flexibility in secondary binding also applies to orthologous transcription factors.

Recently, protein-binding microarray technology has revealed that the forkhead family of transcription factors can acquire novel binding specificity among both orthologs and paralogs (Nakagawa et al. 2013). Importantly, this acquisition seems to have a modular component to it. Some forkhead families can bind both the primary and secondary motif as well as an additional novel motif, whereas others bind to either the primary and secondary or only to novel motifs. It is unknown whether this phenomenon extends to other transcription factor families and the functional consequences of this change.

Here, we investigate orthologous Tbrain (Tbr) transcription factors from the sea star, *Patiria miniata* (*Pm*), and sea urchin, *Strongylocentrotus purpuratus* (*Sp*), to question whether these proteins evolved biochemical changes in their DNA-binding preferences. These proteins were selected as they have well characterized and critical roles in early echinoderm development (Ryan et al. 1998; Shoguchi et al. 2000; Croce et al. 2001; Tagawa et al. 2001; Fuchikami et al. 2002; Horton and Gibson-Brown 2002; Oliveri et al. 2002; Hinman, Nguyen, Cameron, et al. 2003). During sea star embryogenesis, Tbr is highly pleiotropic and required for specification of cell types within the mesoderm, endoderm, and ectoderm (Hinman and Davidson 2007; McCauley et al. 2010). In sea urchins, intriguingly, Tbr appears to have lost these roles and is instead only required for the specification of one type of mesoderm, the skeletogenic mesoderm. These genes are members of the T-box family of transcription factors, which are characterized by having a single T-box DNA-binding domain. The DNA-binding properties of these proteins are relatively well studied. There is a particular interest in understanding how groups of T-boxes with the same primary binding motif, expressed in the same tissue, are capable of exerting distinct functions. Many studies show that these transcription factors are characteristically dose dependent, and others suggest that differences in binding site affinities may be crucial for allowing them to operate in a competitive and hierarchical fashion (Macindoe et al. 2009; Sakabe et al. 2012). Therefore, there is a great interest in understanding the binding properties of these transcription factors.

The echinoderm Tbr proteins are orthologous to vertebrate Eomesodermin (Eomes) (also known as Tbr2), Tbr1, and Tbx21 (Papaioannou and Silver 1998; Croce et al. 2001). As is the case for many vertebrate transcription factors, these paralogs presumably arose as a result of the vertebrate lineage-specific duplication from a single deuterostome ortholog. We show that these three deuterostome orthologs (sea urchin Tbr, sea star Tbr, and mouse Eomes) have a highly similar primary binding motif, which we think has therefore been maintained in the approximately 800 My (Hedges et al. 2006) since these taxa last shared a common ancestor. Here, we show that, the sea star Tbr and mouse Eomes each have a preference for an additional, unique secondary motif, whereas the sea urchin Tbr protein has no preference for a secondary motif. This demonstrates that these orthologs evolved biochemical changes in function of their DNA-binding domains. We show that at saturating levels of Tbr, the primary and secondary motifs are functionally interchangeable in sea stars. The motifs, however, provide different transcriptional responses as Tbr protein levels change. The use of primary and secondary motifs represents a modular component to transcriptional regulation; subsets of target genes under control of secondary motifs can evolve, whereas those regulated by primary motifs remain conserved. Our data indicate that this evolvable function can manifest as differences in relative timing in response to transcriptional state changes. Given the pervasiveness of secondary binding ability among transcription factors, such changes in secondary binding may prove to be an important source of gene regulatory evolutionary change.

## Results

### Sea Urchin and Sea Star Tbr Are Orthologous to Mouse Eomes

**Fig. 1.**
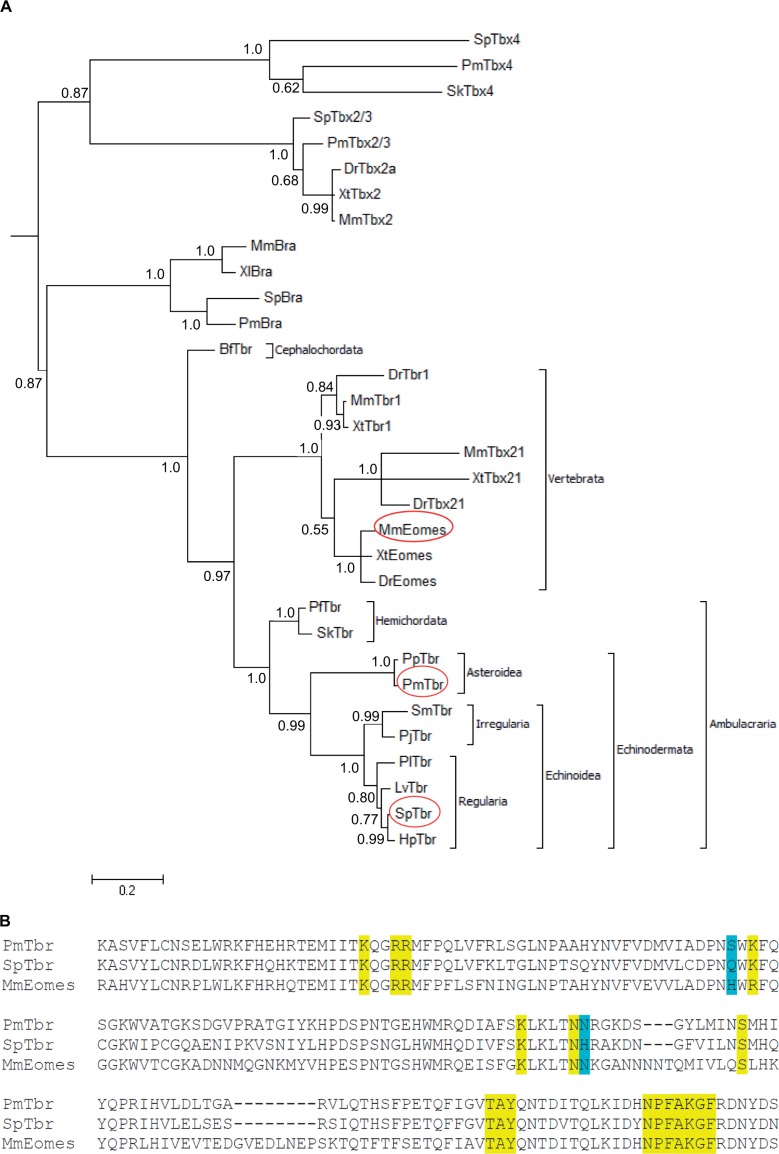
Sequence alignment for *Pm* and *Sp*Tbr Tbox-DNA-binding domains. (*A*) Tree topology was determined using a MrBayes model (TOPALI v2.5) and is based on a character alignment that includes the T-box sequences depicted in supplementary figure S1, Supplementary Material online. Lengths of branches are drawn to the scale indicated (0.2 expected substitutions per site), and the numbers indicate support by posterior probability. *Bf, Branchiostoma floridae*; *Dr*, *Danio rerio*; *Hp*, *Hemicentrotus pulcherrimus*; *Lv*, *Lytechinus variegatus*; *Mm*, *Mus musculus*; *Pf*, *Ptychodera flava*; *Pj*, *Peronella japonica*; *Pl*, *Paracentrotus lividus*; *Pm*, *Patiria miniata*; *Pp*, *Patiria pectinifera*; *Sk*, *Saccoglossus kowalevskii*; *Sm*, *Scaphechinus mirabilis*; *Sp*, *Strongylocentrotus purpuratus*; *Xl*, *Xenopus laevis*; *Xt*, *Xenopus tropicalis*. (*B*) Conceptual translation of *PmTbr*, *SpTbr,* and *MmEomes* T-box domains. Highlighted amino acids indicate residues involved in interaction with DNA according to alignment with *Xl*Bra crystal (Protein Data Bank ID 1XBR) (Müller and Herrmann 1997). Yellow amino acids indicate identical amino acids, whereas blue denotes nonconserved interactions within the echinoderms. Sequence aligments to *Xl*Bra are provided in supplementary figure S1, Supplementary Material online.

In the sea star, *P. miniata*, tbrain (*PmTbr*) was originally isolated from a cDNA library probed with a cDNA clone corresponding to another T-box factor, *PmBrachyury* (*PmBra*) (Hinman, Nguyen, Cameron, et al. 2003). Only *bra* and a single *tbr* ortholog were identified in this screen. To determine whether any other *tbr* orthologs were present within the genome, we bioinformatically queried the *P. miniata* genome sequence (contigs 1.0; Echinobase.org, last accessed July 18, 2014) (Cameron et al. 2009) by performing a tBLASTn identity search to the translated *MmEomes* T-box domain (accession: AK089817.1). We collated the *P. miniata* sequences that matched with an *e* value less than 1e-12. These sequences in turn were used to query the National Center for Biotechnology Information nonredundant protein database using BLASTx (Altschul et al. 1990). Four T-box family members were identified in this comprehensive search. These correspond to a subset of the six T-box family members identified previously in the sea urchin, *S. purpuratus*, genome (Howard-Ashby et al. 2006). We next determined the orthology of these four T-box factors by constructing a gene tree (see Materials and Methods) of these T-boxes and their homologs from other deuterostome animals (fig. 1*A*).

*PmTbr* clusters with a *tbr* gene isolated from another species of sea star (*P. pectinifera*; *PpTbr*), whereas the *SpTbr* clusters with *tbr* orthologs from five other species of sea urchins, including two species of sand dollars, which form a distinct group (Irregularia) within the sea urchins. Importantly, the sea urchin and sea star genes form a single grouping supported by a posterior probability of 0.99. Thus, there is a strong correspondence between the topology of this gene tree and the echinoderm species tree (Pisani et al. 2012). In vertebrates, there are three *tbr* paralogs, namely *eomes*, *tbx21*, and *tbr1*, which also form a single grouping. These three paralogs form a single cluster with the echinoderm orthologs with the node connecting them supported by a posterior probability of 0.97.

Meanwhile, the other T-box proteins isolated in the screen are orthologous to *bra, tbx2/3,* and *tbx4.* Only a single *tbr* ortholog is identified from eight species of echinoderms, including two with sequenced genomes. Therefore, we are confident as reasonably possible that there is a single *tbr* ortholog among these echinoderms and that it is the only echinoderm ortholog of the vertebrate *eomes*, *tbx21*, and *tbr1* paralogs.

### Sea Urchin and Sea Star Tbr Orthologs Have Different DNA-Binding Preferences

The structure and function of transcription factors, especially the DNA-binding domains, are often highly conserved across even widely divergent species. The 180 amino acid T-box domain is particularly well conserved (Macindoe et al. 2009). An alignment of the *Sp*Tbr and *Pm*Tbr DNA-binding domains demonstrate that they are 73% identical and 89% similar (fig. 1*B*). This indicates that these orthologs share high degree of conservation, yet there is variation that could permit functional divergence. We wanted to determine if any of these differences could indeed have a functional consequence. As a first approach, we used the known crystal structure of a closely related T-box protein, *Xenopus laevis* brachyury (*Xl*Bra) (Protein Data Bank ID 1XBR) (Müller and Herrmann 1997) to map the likely DNA contacts within the sea star and sea urchin Tbr amino acid sequences. We also used these sequences to predict the structures of *Pm*Tbr and *Sp*Tbr using the Phyre server (Kelley and Sternberg 2009). The overall structure of the DNA-binding domain is not predicted to be perturbed by the nonidentical amino acids (supplementary fig. 2*A*, Supplementary Material online). Nineteen amino acids are predicted to contact the DNA (highlighted in yellow in fig. 1*B*), and of these, two are not identical between the sea urchin and sea star (blue highlight, fig. 1*B*). At residue 338/428, the *Sp*Tbr protein has a glutamine where *Pm*Tbr has a serine. This appears to be unique for each species as neither is conserved with the residue in *Xl*Bra nor *Mm*Eomes (fig. 1*B*, supplementary fig. S1, Supplementary Material online). However, at residue 389/479, *Pm*Tbr has an asparagine that is also present in vertebrate proteins, whereas *Sp*Tbr has a histidine at this position. Both of these changes occur in residues known to interact with the DNA backbone as opposed to the bases themselves (supplementary fig. S2*B* and *C*, Supplementary Material online). However, in the case of the homeodomain protein, Bicoid, a change in DNA-binding specificity compared with its Antp paralog is correlated with a single backbone-contacting amino acid difference (Hanes and Brent 1989), and so these two changes to Tbr may also impact DNA-binding specificity.

Although suggestive of a potential for a functional difference, protein–DNA interactions are not well understood enough to predict binding preferences. Therefore, it is unclear how these changes and others that do not occur in amino acids that contact DNA might affect specificity for DNA sequences. We therefore sought to determine experimentally if any differences in DNA specificity exist for these orthologs. We bacterially expressed and purified *Pm*Tbr and *Sp*Tbr DNA-binding domains as GST-fusion proteins and used protein-binding microarrays to universally assess their binding preferences (Berger et al. 2006; Berger and Bulyk 2009). It is important to note that these experiments cannot account for the effects that cofactors normally encountered in vivo might have on Tbr DNA-binding specificity. We chose to test only DNA-binding domains because full-length proteins prove to be extremely unstable. In a previously reported study, no difference in DNA binding was observed when full-length and DNA-binding domain versions of *Mm*Tbx5 were compared (Macindoe et al. 2009). Moreover, T-box protein specificity for several homologs, including *Mm*Eomes, has previously been shown to reside in the T-box domain itself, whereas other regions of the protein account for nuclear localization signals and transactivation domains (Conlon et al. 2001). This work suggested that the Tbr DNA-binding domains would be sufficient to capture the full DNA-binding capabilities of these proteins.

Polymerase chain reaction (PCR)-based methods, such as SELEX, have been used to identify consensus sites for other T-box transcription factors (Conlon et al. 2001; Macindoe et al. 2009). However, these experiments, based on technologies available at the time, were limited to identifying only the highest affinity binding motifs. Protein-binding microarrays uncover additional layers of binding specificity, particularly differences in secondary sequence preferences (Badis et al. 2009).

**Fig. 2.**
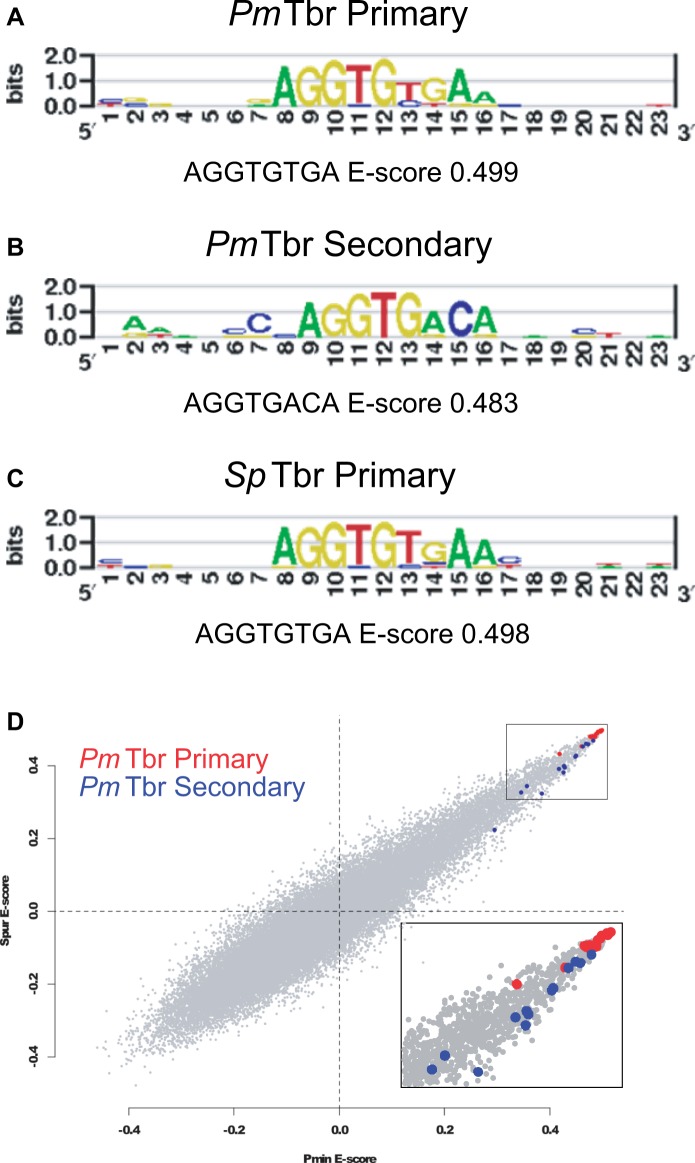
Position weight matrices depicting binding specificities of Tbr orthologs. Position weight matrices represent the top motifs obtained from PBM data using the Seed-and-Wobble algorithm (Berger et al. 2006; Berger and Bulyk 2009) representing *Sp*Tbr and *Pm*Tbr data set 1 (supplementary table S1, Supplementary Material online). Secondary motifs represent high-scoring oligomers whose specificity is not captured by the primary motif. Representative 8-mers and their *E* scores are provided underneath each motif. (*A*) *Pm*Tbr primary binding motif. (*B*) *Pm*Tbr secondary binding motif. (*C*) *Sp*Tbr primary motif. (*D*) Scatterplot of *E* scores for each 8-mer in the *Pm*Tbr versus the *Sp*Tbr data sets. The top 14 8-mer matches to the shared primary position weight matrix are indicated in red, whereas the top 14 matches to the *Pm*Tbr secondary motif are blue. All 8-mers and their reverse compliments (supplementary table S1, Supplementary Material online) were assigned sum probability scores based on how well they matched any 8 bp stretch of *Pm*Tbr primary position weight matrix (from positions 6–17 shown in *A*) and *Pm*Tbr secondary position weight matrix (from positions 7–18 shown in *B*). The 14 matches to each site are the top 0.02% of 8-mer matches ranked by sum probability score. *E* score values indicate the statistical confidence in the seed 8-mer used in position weight matrix construction, where *E* > 0.45 is considered to be a high-confidence binding event (Berger et al. 2006).

The DNA-binding specificity of each Tbr was assayed by protein-binding microarray in duplicate with strong agreement between replicates (*Pm*Tbr Pearson’s *r* = 0.915 and *Sp*Tbr Pearson’s *r* = 0.917). Data sets depicting the *E* score calculated for each 8-mer are available in supplementary table S1, Supplementary Material online. The protein-binding microarray experiments demonstrate that *Pm*Tbr and *Sp*Tbr orthologs recognize the same primary position weight matrix, or motif, which represents the probability of the transcription factor binding to all potential binding sites (fig. 2*A* and *C*). This motif can explain Tbr binding to a large number of 8-mer-binding sites, but for simplicity, it can be represented by the following consensus sequence, 5’-AGGTGTGA-3’. This single binding site was selected for use in subsequent experiments because each position contains the most highly preferred nucleotide predicted by the position weight matrix. Both Tbr orthologs recognize this 8-mer-binding site with a very high *E* score (*Pm*Tbr, *E* = 0.499, *Sp*Tbr, *E* = 0.498). The *E* score (enrichment score) is a nonparametric, modified Wilcoxon–Mann–Whitney statistic developed especially to measure relative binding preference for simple and robust comparison of protein-binding microarray data across data sets (Berger et al. 2006). *E* scores range from −0.5 to 0.5, but scores of 0.45 and greater indicate a stringent binding threshold (Berger et al. 2008; Badis et al. 2009). This motif closely matches previously published T-box consensus sites (Conlon et al. 2001; Macindoe et al. 2009), and in particular, the primary binding site for the mouse ortholog of Tbr, *Mm*Eomes (*E* = 0.497, UniProbe Database), which was also obtained by universal protein-binding microarrays (Badis et al. 2009).

Previous studies using these sensitive protein-binding arrays have shown that approximately 40% of transcription factors that have been tested can bind two distinct motifs (Badis et al. 2009; Gordân et al. 2011). By convention, the motif with the higher seed *E* score is called the primary motif and the next preferred, high confidence motif, the secondary motif. Of our two echinoderm Tbr orthologs, only *Pm*Tbr, however, consistently recognized an additional high *E*-score position weight matrix, best represented by the 8-mer, 5’-AGGTGACA-3’ (*E* = 0.483) (fig. 2*B*, supplementary table S1, Supplementary Material online). Although very similar to the initial motif, it differs in positions 13 and 14, where AC replaces the primary site’s TG. Therefore, here we call the position weight matrix represented by the 8-mer 5’-AGGTGTGA-3’ site the primary motif and that represented by 5’-AGGTGACA-3’, the secondary. These two motifs are not condensed into one more degenerate position weight matrix, because the two distinct motifs better explain the protein-binding microarray data than can a single motif (Badis et al. 2009). This secondary motif was found consistently in replicate experiments. In contrast, *Sp*Tbr never demonstrated strong preference for a particular additional motif (supplementary table S1, Supplementary Material online) over replicate experiments. When we performed a similar analysis using the data from *Sp*Tbr binding to find a secondary motif, the result was simply a more degenerate version of the primary motif. Additionally, we show that *Sp*Tbr and *Pm*Tbr have similar *E* scores for 8-mers that match the primary position weight matrix, but 8-mers corresponding to the *Pm*Tbr secondary motif are preferred by *Pm*Tbr (fig. 2*D*).

The mouse Eomes ortholog also was previously shown to also have two high *E*-score motifs. Although both species of echinoderm and the *Mm*Eomes have highly similar primary position weight matrices, the secondary motifs are dissimilar. The *Mm*Eomes secondary motif is represented as 5’-AGGTGTCG-3’ (*E* = 0.493, UniProbe Database) (Badis et al. 2009). Both *Pm*Tbr and *Mm*Eomes secondary motifs are not the same as the primary motif or each other, particularly in positions 13, 14, and 15 (fig. 2). These data suggest that the primary motif has most likely remained the same over the extensive time scale since these deuterostomes have last shared a common ancestor, whereas the preference for a secondary site has evolved, either through single or multiple losses and gains, over the same time scale. This study is the first demonstration of such an evolutionary change in orthologous transcription factor function.

### *Sp*Tbr and *Pm*Tbr Maintain Similar Affinity for the Conserved Primary Site but Differ Significantly in Their Affinity for *Pm*Tbr’s Secondary Site

**Fig. 3.**
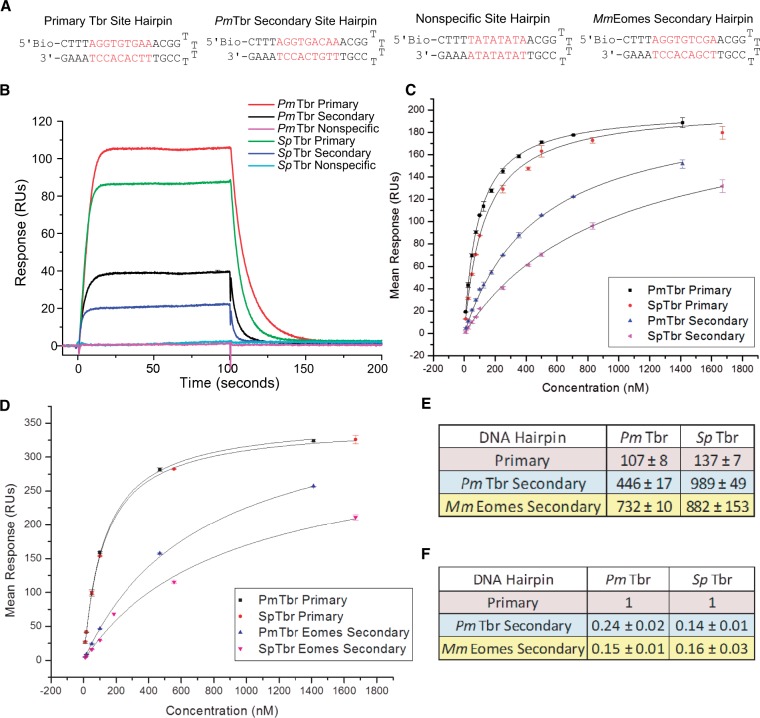
Steady-state affinity evaluations for Tbr DNA-binding domains. (*A*) DNA sequences of oligonucleotide hairpins used in SPR experiments. Nucleotides depicted in red are the predicted protein-binding site. (*B*) Sensorgrams depicting real-time binding of 100 nM *Pm*Tbr and *Sp*Tbr DBD to each biotinylated oligonucleotide. Nonspecific binding was determined using a blank flow cell, which had streptavidin but no DNA bound, and was subtracted from all curves. Equilibrium response (*R*_eq_) was taken from these and curves corresponding to all other protein concentrations at 95 s. Response curves are also buffer subtracted and represent the average of duplicate samples with corresponding error. Results are representative of typical findings from replicate experiments. (*C*) *R*_eq_ versus concentration plus 1:1 binding fits for *Pm* and *Sp*Tbr’s steady-state affinity for primary and *Pm*Tbr secondary binding motifs. Data points indicate the average of duplicate samples plus error from two different concentration series experiments. Errors shown represent standard deviation of data points. (*D*) *R*_eq_ versus concentration plus 1:1 binding fits to determine *Pm* and *Sp*Tbr’s steady-state affinity for *Mm*Eomes secondary binding motif. Primary site binding is also shown because this analysis was performed on a different sensor chip than in C. (*E*) Dissociation constants of each Tbr for each oligonucleotide plus standard error of the mean. (*F*) Relative affinity for each ortholog for each DNA Hairpin plus standard error of the mean. All values are relative to the ortholog’s affinity for the primary site. *K*_D_s indicate average for two experimental runs, both of which were performed with duplicate scrambled concentration series, with the exception of primary binding site values, which come from data depicted in (*C*) and (*D*), and therefore include more experiments.

Given that the functional amino acids that differ between *Pm*Tbr and *Sp*Tbr involve backbone contacts, we next used surface plasmon resonance (SPR) to determine the affinities that *Pm*Tbr and *Sp*Tbr had for each of the identified motifs. Biotin-labeled oligonucleotides were designed to fold into a hairpin containing either the primary site, the *Pm*Tbr secondary site, the *Mm*Eomes secondary site, or a nonspecific site that was found to be poorly bound by both Tbr orthologs in the protein-binding microarray data (*Pm*, *E* = −0.03, *Sp*, *E* = −0.04) (fig. 3*A*).

Protein association and dissociation, which occur when each protein flows across the sensor chip and when wash buffer removes bound protein, respectively, are depicted as sensorgrams (fig. 3*B*). A comparison of this binding response at 100 nM Tbr DNA-binding domain on each oligomer reveals that neither protein binds the nonspecific site (fig. 3*B*). Additionally, the shape of the sensorgrams indicates that stable equilibrium is reached quickly, and, therefore, equilibrium response can be ascertained and used to calculate affinity.

To determine affinities, equilibrium response units (RUs) were taken at 95s into the association phase, where equilibrium is established, as indicated by the slope = 0 in the sensorgrams (fig. 3*B*). Such measurements were taken from sensorgrams corresponding to at least five, but as many as ten, concentrations. Samples of Tbr from each species were applied to the same SPR chip alternately so both proteins were assayed with equal binding conditions. The equilibrium RU values were plotted versus protein concentration and fit to a 1:1 binding model (Adjusted *R*^2^ > 0.99) (fig. 3*C* and *D*). Averaged affinity results from four or more experiments across these protein concentrations are shown in figure 3*E*. *Pm*Tbr recognizes the primary motif slightly better than does *Sp*Tbr, with affinities of 107 ± 8 nM for *Pm*Tbr and 137 ± 7 nM for *Sp*Tbr. By comparison, *Pm*Tbr binds the secondary site with significantly greater affinity that does *Sp*Tbr. *Pm*Tbr binds the secondary site with an affinity of 446 ± 17 nM and *Sp*Tbr binds with an affinity of 989 ± 49 nM (two-tailed *t*-test, *t* = 11.612, df = 6, *P* = 0.0007) (fig. 3*C* and *E*). Neither echinoderm Tbr ortholog binds particularly well to the *Mm*Eomes secondary site; PmTbr binds with an affinity of 732 ± 10 nM and SpTbr with an affinity of 882 ± 153 nM (fig. 3*D* and *E*).

We also compared relative affinity of *Pm*Tbr and *Sp*Tbr for each secondary site versus affinity for the primary site (fig. 3*F*) by dividing their respective primary site *K*_D_ by *K*_D_s for all other binding sites. This allowed us to ascertain whether *Sp*Tbr’s lower affinity for the secondary site could be due to an overall reduction in binding affinity because even *Sp*Tbr’s affinity for the primary site is slightly lower than *Pm*Tbr’s. The relative affinity of the secondary site versus the primary site is 0.24 for *Pm*Tbr, whereas for *Sp*Tbr, it is significantly lower at 0.14 (two-tailed *t*-test, *t* = 8.944, df = 6, *P* = 0.00022, Bonferroni corrected). *Sp*Tbr’s relative affinity for *Pm*Tbr’s secondary site is comparable to the relative affinity both Tbrs have for *Mm*Eomes’s secondary site (0.15 and 0.16). *Pm*Tbr clearly binds its own secondary site better than it binds the *Mm*Eomes secondary site (two-tailed *t*-test, *t* = 8.165, df = 4, *P* = 0.0024, Bonferroni corrected). It also has a stronger relative affinity for this site than *Sp*Tbr has for the secondary site from either *Pm*Tbr or *Mm*Eomes.

The data shown in figure 3 provide an independent confirmation of the protein-binding microarray data (fig. 2 and supplementary table S1, Supplementary Material online) with an additional quantification of sequence affinity. They show that *Pm*Tbr has a stronger preference for its secondary motif than does *Sp*Tbr in spite of the similar affinities these echinoderm proteins have for their primary motif and for the *Mm*Eomes secondary motif. Although *Sp*Tbr tends to bind all tested sites with slightly less affinity than does *Pm*Tbr, it is notable that this is not enough to explain the larger difference in binding observed for the *Pm*Tbr secondary site, as demonstrated by comparisons of relative affinity.

### The Secondary Site Can Substitute for the Primary Site In Vivo When Tbr Levels Are High but Not When They Are Reduced

**Fig. 4.**
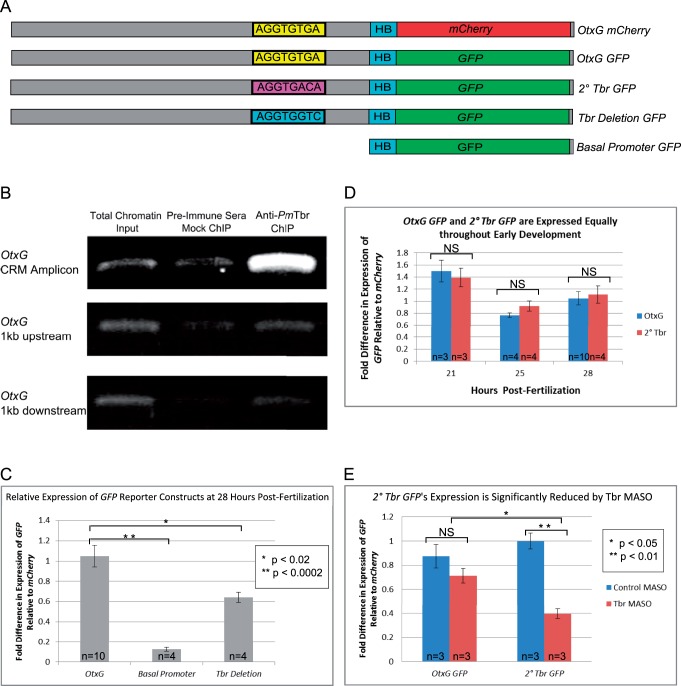
*Pm*Tbr can use the primary and secondary sites in vivo to drive reporter gene expression interchangeably except when Tbr levels are reduced. (*A*) Schematics depicting *OtxG mCherry*, *OtxG GFP*, *2 ° Tbr GFP*, *Tbr Deletion GFP*, and *Basal promoter GFP* reporter gene constructs including the endogenous and mutated Tbr-binding motifs of interest. (*B*) ChIP PCR using primers pairs surrounding *OtxG* (*OtxG* CRM Amplicon) or primers pairs 1 kb up or downstream of *OtxG*. EtBr-stained gel shows amplicons obtained from total chromatin, preimmune sera mock ChIP, and Anti-*Pm*Tbr ChIP. (*C–E*) qPCR analysis of GFP expression levels driven by constructs indicated. All *GFP* expression levels have been normalized to *mCherry* levels that were driven by the coinjected *OtxG mCherry* construct. (*C*) Normalized GFP expression levels of *OtxG GFP*, *Basal Promoter GFP*, and *Tbr Deletion GFP* at 28 h. (*D*) At developmental time points 21 h, 25 h, and 28 h, Tbr is equally able to drive expression from *OtxG* reporters containing an endogenous primary site and introduced secondary site. The normalized expression level of *GFP* in *OtxG GFP* (blue bars) compared with *2 ° Tbr GFP* (red bars) is not significantly different. (*E*) Normalized *GFP* expression levels resulting from *2 ° Tbr GFP* or *OtxG GFP* coinjected with control MASO (blue bars) or Tbr (red bars) MASOs. In panels, *n* indicates the number of replicate samples, each consisting of 50 sibling embryos. All error bars indicate standard error of the mean. *P* values indicate the results of a two-tailed *t*-test. Details of these tests are provided in the main text. NS indicates not significant by two-tailed *t*-test.

We next wanted to determine how the primary and secondary sites function in vivo to regulate transcription to understand whether these differences are biologically relevant. We had previously characterized a CRM (*OtxG*) that controls the expression of the sea star *otx* gene (Hinman et al. 2007) and contains a single endogenous Tbr site that is a perfect match to the protein-binding microarray-derived primary motif (fig. 4*A*). We first confirmed that Tbr binds directly to this CRM in vivo using chromatin immunoprecipitation (ChIP) PCR. ChIP was performed in embryos at 30 hours post-fertilization (h), a time point during which *OtxG* is known to be active (Hinman et al. 2007). We show that the genomic region containing *OtxG* is greatly enriched in chromatin pulled down by the anti-*Pm*Tbr antibody compared with input chromatin and mock ChIP chromatin (fig. 4*B*). Importantly, genomic regions 1 kb up or downstream of *OtxG* are not enriched in *Pm*Tbr ChIP DNA (fig. 4*B*).

We next produced a series of constructs to determine how the primary and secondary motifs would behave in vivo (fig. 4*A*). “*Basal Promoter GFP*” is a previously existing construct that contains only a basal promoter in a *GFP* expression vector (Hinman et al. 2007). This imparts very low levels of ubiquitous *GFP* expression. The “*OtxG GFP*” construct has the endogenous *OtxG* CRM added upstream of the basal promoter. “*2 ° Tbr GFP*” has a 2-bp mutation which changes the endogenous primary motif to a secondary motif. “*Tbr Deletion GFP*” ablates the Tbr-binding site by changing the same bases mutated in “*2 ° Tbr GFP*” but so that the resulting site is one that had an average *E* score of −0.058 in the protein-binding microarray data set. By comparison, our motifs selected to represent the primary and secondary position weight matrices had average *E* scores of 0.499 and 0.483, respectively. *Pm*Tbr should, therefore, be unable to bind this site.

These constructs are injected into embryos where they express the reporter gene in clones of cells. In each experiment, our various *GFP* constructs are coinjected with *OtxG mCherry*, which is identical to *OtxG GFP* except that coding sequence for the *mCherry* gene replaces that of the *GFP* reporter. The *OtxG mCherry* construct is used to normalize each sample for differences in injection volume, mosaicism of reporter incorporation, and embryo collection and processing. We used *mCherry* rather than an endogenous housekeeping gene to normalize *GFP* expression levels as this reporter will also account for injection variation. We do expect that there may be some differences in overall *GFP* versus *mCherry* transcript levels driven by identical CRMs because these mRNA transcripts may have different stability in vivo. It is important to note, however, that none of our assays directly compares *GFP* to *mCherry* levels but instead compare *GFP* levels across assays at a single time point that have been normalized to *mCherry*. Therefore, absolute differences in coinjected reporter levels themselves will not affect our analyses.

**Fig. 5.**
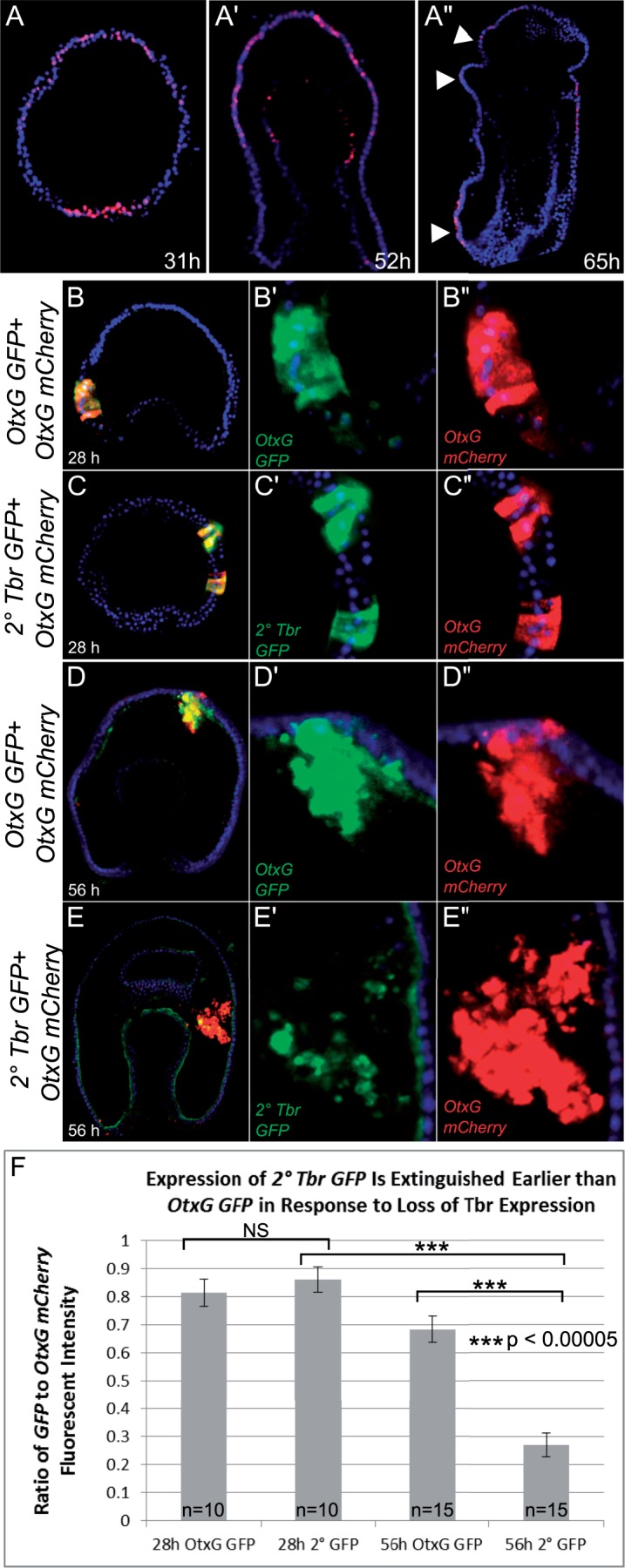
Secondary Tbr reporter has reduced expression compared with OtxG in the ectoderm when Tbr levels are declining. (*A–A*″) In all panels, blue indicates DAPI nuclear stain and red indicates Tbr localization. (*A*) Thirty-one hours blastula stage *Patiria miniata* embryo; (*A*′) 52-h gastrula stage embryo; and (*A*″) 65-h late gastrula stage embryo. Arrow heads indicate localization, which is present in only the ciliary band ectoderm by 65 h. (*B–E*″) In all panels, blue indicates DAPI nuclear stain, red indicates *mCherry* transcripts labeled by CyIII, and green indicates *GFP* transcripts labeled by fluorescein. (*B*), (*C*), (*D*), and (*E*) depict the entire embryo with merged expression, whereas (*B*′–*B*″), (*C*′–*C*″), (*D*′–*D*″), and (*E*′–*E*″) are insets of the region of interest for each probe. (*B–C*″) *OtxG GFP* and *2 ° Tbr GFP* both coexpress spatially with *OtxG mCherry* at 28 h (*D–D*″). *OtxG GFP* reporter coinjected with *OtxG mCherry* at 56 h. The reporters are still spatially coexpressed at this stage. (*E–E*″) *2 ° Tbr GFP* reporter coinjected with *OtxG mCherry* at 56 h. *GFP* expression is reduced compared with *OtxG GFP,* whereas *mCherry* levels remain more consistent. (*F*) Quantification of fluorescent intensities of fluorescein (*GFP*) relative to CyIII (*mCherry*) at 28 h and 56 h. *N* indicates the number of embryos imaged. Error bars indicate standard error of the mean. *P* values indicate the result of two-tailed *t*-tests, which are described in the Results.

We assayed the expression of these reporter genes using a combination of approaches. Quantitative reverse transcription PCR (qRT-PCR) was used to determine the abundance of the reporters relative to each other (fig. 4). Fluorescent whole-mount in situ hybridization (FISH) was used to examine the spatial localization of these reporters (fig. 5). We use FISH rather than assays for fluorescent protein localization, as RNA localization is a more direct measure of transcript regulation and should coincide with qRT-PCR. GFP and mCherry proteins are relatively stable and can persist within the embryo after gene expression is extinguished. We also quantified fluorescent signal strength in whole-mount FISH embryos using ImageJ (Schneider et al. 2012). This last approach allows us to specifically estimate the abundance of each reporter within a particular spatial location (fig. 5).

We first performed a series of controls to verify the utility of this reporter system. We confirmed that the *Basal Promoter GFP* construct does not drive significant expression on its own when coinjected with other constructs. *Basal Promoter GFP* drives expression at a roughly 10-fold lower level than *OtxG GFP* in sibling embryos of the same stage (28 hours post-fertilization (h)). This indicates that there is no cross-regulation between the *OtxG mCherry* construct used for normalization and the *Basal Promoter GFP* coinjected constructs (two-tailed *t*-test, *t* = 9.082, df = 12, *P* = 0.0002, Bonferroni corrected) (fig. 4*C*). *Tbr Deletion GFP* expression is also significantly reduced compared with *OtxG GFP*, indicating that the Tbr-binding site within *OtxG* is crucial for normal expression levels (two-tailed *t*-test, *t* = 3.305, df = 12, *P* = 0.011. Bonferroni corrected). Combined, these experiments establish that the validity of this reporter system for assaying primary and secondary site usage in vivo. They demonstrate that the basal promoter does not drive any significant expression when coinjected with other constructs and that the Tbr site is a functional in vivo binding site.

We then compared the expression driven by our primary and secondary sites using this reporter system. Tbr levels are very high maternally and throughout early development as shown by western blot (supplementary fig. S3*A*, Supplementary Material online). Using qRT-PCR, we show that *2 ° Tbr GFP* and *OtxG GFP* drive expression at roughly the same levels in vivo at three early developmental time points; 21 h (two-tailed *t*-test, *t* = 0.404, df = 4, *P* = 0.650), 25 h (two-tailed *t*-test, *t* = 1.505, df = 6, *P* = 0.148), and 28 h (two-tailed *t*-test, *t* = 0.296, df = 12, *P* = 1, Bonferroni corrected) (fig. 4*C*). These data, therefore, convincingly show that Tbr is able to use the secondary site in place of the naturally occurring primary site in vivo and with no significant change in transcription of the reporter. This suggests that at these time points, there are sufficient levels of Tbr present to overcome the differential affinity for these sites, and therefore, Tbr binds either the primary or secondary site interchangeably to drive gene expression.

We next sought to determine whether the Tbr protein could differentiate between these sites when protein levels are reduced. To this aim, we coinjected each construct with either 400 µM control morpholino antisense oligonucleotide (MASO) or *Pm*Tbr-specific translation blocking MASO. These modified oligonucleotides bind in a sequence specific manner to the translation start site of the transcript to block translation and have been used successfully in previous work from our lab (Hinman et al. 2007; McCauley et al. 2010). At this concentration, the Tbr MASO drastically reduces, but does not eliminate, Tbr protein. Knock-down efficiency of all samples was confirmed by assaying for changes in expression of known Tbr target genes, *otxβb* and *delta*, by qRT-PCR (supplementary fig. S3*B*, Supplementary Material online) (Hinman and Davidson 2007). Therefore, we are confident that our Tbr MASO is reducing levels of Tbr protein. In a Tbr knockdown, *2 ° Tbr GFP* drives expression at 40% the level of its expression in sibling Control MASO embryos at 28 h (two-tailed *t*-test, *t* = 6.360, df = 4, *P* = 0.0067, Bonferroni corrected) (fig. 4*E*). To control for any effects that might be associated with the different reporters in this experiment, we show that at 28 h, normalized expression of *OtxG GFP* is not significantly different between Tbr MASO and sibling Control MASO embryos (two-tailed *t*-test, *t* = 1.410, df = 4, *P* = 0.334, Bonferroni corrected). Furthermore, when we consider the expression of *OtxG GFP* compared with *2 ° Tbr GFP* when they are expressed in Tbr MASO embryos (fig. 4*E*, comparison between red bars), *2 ° Tbr GFP* is expressed at significantly lower levels (two-tailed *t*-test, *t* = 3.880, df = 4, *P* = 0.022, Bonferroni corrected). This demonstrates that even though the *2 ° Tbr GFP* construct differs from *OtxG GFP* by only 2 bp, it is significantly more sensitive to Tbr knockdown than is *OtxG GFP*. This indicates that the secondary binding site is more sensitive to in vivo protein levels, as predicted from the in vitro affinity data.

### The Secondary Site Responds Faster to Tbr’s Endogenous Temporal Gradient

We wanted to determine whether the secondary and primary binding sites would respond differently to endogenously changing levels of Tbr. To test how the primary and secondary sites might differ in their response to a temporal decline in Tbr levels, we first determined when Tbr decreases endogenously. Tbr levels are high maternally, which makes it difficult to determine how genes respond to zygotic Tbr levels as the gene’s transcription is initiated (supplementary fig. S3*A*, Supplementary Material online). However, we see that during the later gastrula stages, between 54 h and 65 h, Tbr goes from being localized broadly throughout the ectoderm (31 h and 52 h embryos) to being specifically localized within the ciliary band territory within the ectoderm (fig. 5*A*). We also see an overall reduction in Tbr levels between 48 h and 70 h by western blot (supplementary fig. S3*A*, Supplementary Material online). The *otx* gene, regulated by Tbr through the *OtxG* CRM, has a similar progression of its expression domain and time course (Hinman, Nguyen, and Davidson 2003).

We therefore determined whether expression driven by the *2 ° Tbr GFP* reporter extinguishes more rapidly in the ectoderm between 54 h and 65 h than that driven by *OtxG*. We examined the expression of GFP and mCherry reporters using FISH. In all of these stages, endoderm expression of Tbr is high (Hinman, Nguyen, Cameron, et al. 2003), which necessitates spatial comparison of transcripts localized to the ectoderm as opposed to qRT-PCR, which can only determine global transcriptional levels. We examined the spatial coexpression of *GFP* and *mCherry* in appropriately staged embryos and then quantified levels of expression in these cells. As in our qRT-PCR experiments, we normalize the level of *GFP* expression driven by *OtxG GFP* and *2 ° Tbr GFP* to *mCherry* levels driven by *OtxG mCherry*. We first confirmed that *OtxG GFP* and *OtxG mCherry* coexpress in the same cells in early (28 h, fig. 5*B*–*B*″) and late development (56 h, fig. 5*D*–*D*″), so that *mCherry* expression can be used for normalization of fluorescent intensity. We next show that *2 ° Tbr GFP* and *OtxG mCherry* also coexpress in the same set of cells at these time points (fig. 5*C*–*C*″ and 5 *E*–*E*″). Finally, we quantify and compare the normalized *GFP* expression driven by primary and secondary motifs in early development (28 h) when Tbr levels are high and in late development (56 h) when Tbr levels are low.

At 28 h, we show that *OtxG GFP* does not drive significantly different expression in the ectoderm compared with *2 ° Tbr GFP* (two-tailed *t*-test, *t* = 0.663, df = 18, *P* = 0.987, Bonferroni corrected). Thus, at this stage, as predicted by our earlier quantitative assays, there is no effect of primary versus secondary binding site on the abundance of reporter gene expression, and we also show here on spatial localization. When we compare the expression of *2 ° Tbr GFP* to *OtxG GFP* at 56 h, however (compare ratio of *E*′/*E*″ to *D*′/*D*″; fig. 5*E*), we find that *2 ° Tbr GFP* reporter is expressed in reduced patches and at visually lower levels. Quantification of fluorescent intensities of normalized *GFP* signals demonstrates significant reduction of *2 ° Tbr GFP* expression relative to *OtxG GFP* (two-tailed *t*-test, *t* = 6.109, df = 28, *P* = 0.0000019, Bonferroni corrected). These data (figs. 4 and 5) show that a 2 bp change from the higher affinity primary to the lower affinity secondary Tbr binding site is sufficient to elicit a response to reduced Tbr levels that is more pronounced than the wild-type response.

## Discussion

There has been a great deal of interest and controversy surrounding theories of how developmental GRNs might evolve. Debate has centered on the effects that protein versus *cis*-regulatory mutations may have on the capacity for change in a GRN. Much work suggests that CRM variation is the prominent source of change to GRNs and evolution of novel phenotypes (reviewed in Wray 2007; Rebeiz and Williams 2011; Wittkopp and Kalay 2012; Rubinstein and de Souza 2013). There are many explanations for why CRMs are so equipped to evolve, but a crucial source of their evolutionary flexibility is their modularity. A single gene is frequently regulated by many CRMs, each CRM orchestrating expression of that gene in a specific spatiotemporal context (Arnone and Davidson 1997). So then, a particular CRM for a given gene can be lost, gained, or altered independently from all of the other CRMs, and likewise, binding sites within a CRM can be lost, gained, or altered independently from the rest of the sites within the CRM. These properties create a scenario with very little pleiotropy and as a result, a great deal of evolutionary freedom.

A key to understanding how protein changes can affect GRNs therefore is to understand the ways that proteins can themselves evolve in ways that reduce pleiotropy. In actuality, proteins are often composed of multiple domains, which may be gained, lost, and changed independently of each other to create diverse proteins (Levitt 2009; Wang and Caetano-Anollés 2009; Kersting et al. 2012). Each domain has the capacity to be modified individually, and some of these modifications may limit the activity of the protein to a specific time and place. A novel protein–protein interaction, for example, might limit the activity of a protein to contexts where it is coexpressed with its new cofactor. It is unsurprising then that changes in protein–protein interactions (Löhr and Pick 2005; Tuch et al. 2008) and post-translational modifications (Lynch et al. 2011) also allow for the evolution of novel features and rewiring of GRNs.

Understanding of how transcription factors might directly evolve changes in DNA-binding properties has been less clear. Outside of a few striking examples (Hanes and Brent 1989; Baker et al. 2011; Nakagawa et al. 2013), it has been considered that this feature of transcription factor function will remain highly conserved and will not represent a substantial source of evolutionary novelty. Recent work, however, demonstrates that DNA-binding properties also have a capacity to be modular as they can have secondary or alternative binding preferences in addition to their primary or most preferred binding site (Badis et al. 2009; Gordân et al. 2011; Busser et al. 2012; Nakagawa et al. 2013). Other work reveals that transcription factors need multiple binding sites that differ in affinity because they are crucial for executing unique developmental functions (Rowan et al. 2010; Peterson et al. 2012). In the *Drosophila* mesoderm, many homeodomain transcription factors are coexpressed and share a primary binding motif. Use of secondary binding sites, which are unique to a particular paralog, allows different homeodomain paralogs to bind appropriate CRMs and execute discrete developmental functions (Busser et al. 2012). The ability to use multiple binding site sequences imparts flexibility in gene regulation and is crucial for developmental functions of these transcription factors. Several surveys of transcription factors indicate that secondary binding preferences are common and frequently differ between paralogous transcription factors (Badis et al. 2009; Gordân et al. 2011). Paralog diversity, however, represents an evolutionary scenario particular to gene duplication events. A pair of paralogs originates from a single protein, and, therefore, they are often able to divide the responsibilities of the original protein between them. In some cases, one paralog maintains all the functions of the original protein and the other is free to neofunctionalize (Plaitakis et al. 2003; Zhang et al. 2004; Lee and Irish 2011). In either case, this division of labor relieves evolutionary constraint on one or both paralogs and may allow new secondary binding preferences to evolve.

Here, we demonstrate for the first time that orthologous transcription factors also diversify by evolving differences in secondary motif binding. We show that the two echinoderm Tbr orthologs, *Sp*Tbr and *Pm*Tbr, bind a highly similar primary motif. This motif also matches the previously published primary motif of *Mm*Eomes (Badis et al. 2009). *Sp*Tbr and *Pm*Tbr recognize that motif with similar affinity. Importantly, we determine that there is a greater evolutionary variation in secondary binding motif preference since echinoderms and vertebrates last shared an ancestor. We find that *Pm*Tbr and *Mm*Eomes recognize distinct secondary motifs, whereas the sea urchin *Sp*Tbr does not have any significant secondary motif preference and has a significantly reduced ability to bind *Pm*Tbr and *Mm*Eomes’s secondary motifs.

The fold changes in binding site affinity that we determine here between preferences for the sea star primary and secondary motifs are the same order of magnitude as observed between different classes T-box transcription factors for a consensus primary site. For example, Macindoe et al. (2009) determined the affinities that three divergent T-box proteins, human Tbx5 (*Hs*Tbx5), Mouse Tbx20 (*Mm*Tbx20), and human Tbx2 (*Hs*Tbx2), had for their consensus primary sequence, AGGTGTGA. This work demonstrated that *Mm*Tbx20, *Hs*Tbx5, and *Mm*Tbx2 bound to this site with affinities of 913 nM, 232 nM, and 1,511 nM, respectively. It was suggested that this difference in affinity, which is less than 2-fold between *Mm*Tbx20 and *Mm*Tbx2, could be functionally significant and permit the competitive, hierarchical gene regulation known to occur when these transcription factors are coexpressed in the developing heart (Macindoe et al. 2009).

This study is the first demonstration of this type of evolutionary change in orthologous transcription factor function. This finding points to a previously overlooked source of modularity for evolution to exploit and, therefore, to a mechanism for allowing a transcription factor to evolve a new function. We speculate that *Pm*Tbr may be able to carry out multiple developmental functions simultaneously by dividing them among its two binding motifs. *Pm*Tbr is needed for the correct specification of endoderm, mesoderm, and ectoderm during sea star embryogenesis (Hinman and Davidson 2007; Hinman et al. 2007; McCauley et al. 2010). Meanwhile, *Sp*Tbr has a single role in the sea urchin embryo, which is to specify skeletogenic mesenchyme (Croce et al. 2001; Oliveri et al. 2002). Even within the skeletogenic network, *Sp*Tbr has relatively few inputs into skeletogenic genes (Rafiq et al. 2012) suggesting that it is a much less pleiotropic gene than *Pm*Tbr. In hemichordates and cephalochordates, the Tbr ortholog is also expressed in multiple embryonic tissue types, including endoderm and ectoderm (Tagawa et al. 2001; Horton and Gibson-Brown 2002), suggesting that these orthologs and *Pm*Tbr may share an ancestral function in the endoderm and ectoderm that must have been lost in sea urchins.

The ability to divide functions between different binding motifs has potential to be very useful during development because a limited number of regulatory molecules must orchestrate the specification of an increasingly complex embryo. Ideally, such regulatory molecules will be as multifunctional as possible to allow development to progress rapidly and create diverse cell types. Yet, this pleiotropy is what causes transcription factors to be evolutionarily constrained. Our finding that these functions can be uncoupled and evolve independently through separate binding sites offers a mechanism by which new features can arise.

**Fig. 6.**
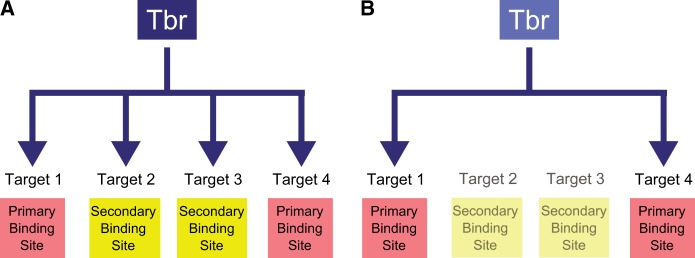
Modular binding of Tbr may allow for diverse transcriptional responses during development and allow for greater evolvability. (*A*) When *Pm*Tbr levels are high, transcription of target genes can be activated via primary and secondary sites. Activated targets are denoted by arrow inputs. However, when *Pm*Tbr levels are low (*B*), only genes regulated via primary sites are activated, whereas those that use secondary sites will have no or reduced transcription, which are shown with no arrows. Because *Sp*Tbr has reduced affinity for the secondary site, it will encounter the later scenario, shown in (*B*), more frequently and may never have an opportunity to activate target genes that are dependent on secondary sites.

We also demonstrate that the secondary binding site is more responsive to changes in Tbr protein levels during development. This quality is particularly important for functions that require rapid transcriptional responses and may be especially important during early development where the timing of developmental events must be precisely coordinated. We predict such affinity differences are also advantageous when a rapid transcriptional response is required during development for some but not all target genes (fig. 6). Such targets can make use of more sensitive, lower affinity secondary sites.

It is often assumed that transcription factors are under an enormous amount of evolutionary constraint because they regulate large numbers of target genes. Presumably, these targets are essential to the organism and must be maintained by all orthologs that arise by speciation. However, if these target genes are subdivided into groups based on the binding sites they are regulated by, then there are fewer genes affected by changes in binding preference. This reduces pleiotropy, because a loss of ability to use a secondary site would affect only a subset of target genes, whereas others would be regulated normally (fig. 6). *Sp*Tbr should be able to maintain developmental functions associated with the primary site, yet its reduced ability to utilize a secondary site may have led to evolutionary differences in cell patterning and specification between these species. This modification in function between orthologs will not only lead to a dramatic loss or gain of target genes but also offers a mechanism to affect timing control of gene regulation. Change in relative order or timing of developmental events can be acquired by evolving higher or lower affinity for a secondary binding site. We hypothesize that this newfound source of modularity in orthologous transcription factors offers a previously overlooked source of GRN evolutionary change.

## Materials and Methods

### Phylogenetics

Tbr orthology was established using a MrBayes model (JTT plus Gamma), 5 runs, 100,000 generations, sampling frequency of 10, in TOPALi v2.5 (Milne et al. 2004). Branches are supported by posterior probability. The T-box domain alignment of all represented proteins was generated by Clustal Omega (Sievers et al. 2014) and is shown in supplementary figure S1, Supplementary Material online. Accession numbers are listed in supplementary figure S1, Supplementary Material online.

### Protein Expression and Purification of DNA-Binding Domains

GST fusion protein constructs for protein-binding microarray, and SPR were made by cloning T-box sequences into pKM vector and were purified from BL21 *E. coli*. The T-box domain constructs consisted of residues 272–466 of *Pm*Tbr and residues 362–554 of *Sp*Tbr to include the whole T-box plus five amino acids flanking each side. Cultures were grown at 20 °C, and protein expression was induced by addition of 0.2 mM IPTG at OD600 0.5, and growth was continued overnight. Cell pellets were resuspended in PBS Triton x-100 (0.1% v/v) (pH 7.5) for protein-binding microarrays or 20 mM Mops (pH 7.5), 150 mM NaCl, 1 mM DTT, and 0.005% Surfactant P20 (v/v) for SPR. In both cases, complete protease inhibitors (Roche Diagnostics, Indianapolis, IL) were added just prior to use, and cells were lysed by sonication. All fusion proteins were purified by GSH affinity chromatography (Thermo Scientific Pierce, Rockford, IL). For protein-binding microarray experiments, glycerol was added to eluted proteins to 10% (v/v), and single-use aliquots were flash-frozen and stored at −80 °C. For SPR protein samples, T-box DNA-binding domains were cleaved from GST-His on beads by treatment with TEV protease (Eton Bioscience, San Diego, CA). DNA-binding domains were then flash frozen and stored at −80 °C in single-use aliquots.

### Protein-Binding Microarrays

Custom-designed, “universal” oligonucleotide arrays (Agilent Technologies, AMADID #016060 [Zhu et al. 2009]) were converted to double-stranded DNA arrays by primer extension and used in protein-binding microarray experiments essentially as described previously (Berger et al. 2006); 200 nM samples of *Pm*Tbr and *Sp*Tbr were assayed in PBS (pH 7.5). Two replicate data sets for each protein are reported in supplementary table S1, Supplementary Material online. Microarrays were scanned and quantified and then analyzed using the Universal PBM Analysis Suite and the Seed-and-Wobble motif derivation algorithm as described previously (Berger et al. 2006; Berger and Bulyk 2009).

### Surface Plasmon Resonance

The sequences of 5’Biotin-labeled hairpin DNA oligomers are depicted in figure 3*A*; 25 nM stocks of hairpin oligomers were diluted in HBS-EP buffer (0.01 M HEPES, pH 7.4, 0.15 M NaCl, 3 mM ethylenediaminetetraacetic acid [EDTA], 0.005% Surfactant P20). These were applied to a streptavidin-coated CM5 chip, prepared according to Nguyen et al. (2006), with minor modifications for a Biacore T100 SPR instrument. The first flow cell was left blank for reference subtraction, whereas primary, secondary, and nonspecific DNA hairpins were immobilized to flow cells 2–4, respectively, such that each had 150 RUs of DNA. Separate chips were made to assess affinity for *Pm*Tbr secondary and *Mm*Eomes secondary sites (both on flow cell 3 of their respective chips). Both chips were designed with the primary site hairpin on flow cell 2 and nonspecific hairpin on flow cell 4. Because the maximal binding capacity of each chip was not equivalent, this necessitated that the data shown in figure 3*C* and *D* be split into separate graphs. The sensor chip was washed several times in running buffer prior to use (50 mM Mops, 150 mM NaCl, 1 mM DTT, and 0.01% [vol/vol] P20 surfactant). Kinetic measurements were performed at 20 °C with a flow rate of 30 μl/min. Tbr DNA-binding domain protein samples were run alternately across the same chip, and all four flow cells were exposed to a sample simultaneously. The concentration series was scrambled for each protein. Immediately following protein injection, buffer was injected to monitor dissociation. Zero concentration (buffer only) samples were included and used to subtract background from protein samples. Data were analyzed first using the BIAevaluation software to determine steady-state response levels for each concentration 95 seconds after injection start. These data were then evaluated using Origin and a 1:1 binding model to determine *K*_D_s.

### Embryo Culture and Injection

*Patiria miniata* embryos were obtained and injected as described in Hinman, Nguyen, Cameron, et al. (2003) and Cheatle Jarvela and Hinman (2014).

### Reporter Expression Constructs

*OtxG GFP* and *Basal promoter GFP* reporter constructs were developed by Hinman et al. (2007). *2 ° Tbr GFP*, *Tbr Deletion GFP*, and *OtxG mCherry* were developed from these existing constructs using the methods described in Hinman et al. (2007). Primer sequences are provided in supplementary table S2, Supplementary Material online.

### Fluorescent Whole-Mount *In Situ* Hybridization

FISH was performed as previously described (Yankura et al. 2010) using digoxigenin- or dinitrophenol-labeled antisense RNA probes targeted to *GFP* and *mCherry,* respectively. Samples consisted of cohorts of sibling embryos injected with either *OtxG GFP* plus *OtxG mCherry* or *2 ° Tbr GFP* plus *OtxG mCherry*. Embryos were reared at 15 °C until 28 h or 56 h.

### Image Analysis

FISH embryos were imaged with a Carl Zeiss LSM-510 Meta DuoScan Inverted Confocal Microscope. Laser power, gain, and digital offset settings were optimized for embryos injected with *OtxG GFP* plus *OtxG mCherry* and then left unchanged for subsequent imaging of sibling embryos injected with *2 ° Tbr GFP* plus *OtxG mCherry*. The relative fluorescence of *mCherry* transcripts (CyIII) to *GFP* transcripts (fluorescein) was quantified using ImageJ (National Institutes of Health, Bethesda, MD). All images were background subtracted using “BG subtraction from ROI” plugin prior to analysis. The “Measure” function was used to determine the mean fluorescence value of a region in interest for both channels.

### Quantitative RT-PCR

Total RNA from injected embryos was obtained using GenElute Mammalian Total RNA kit (Sigma, St. Louis, MO). The total RNA was used to make cDNA using iSCRIPT Select cDNA synthesis kit (Bio-Rad, Hercules, CA). Quantitative RT-PCR (qRT-PCR) was performed according to Hinman, Nguyen, Davidson (2003) using an Applied Biosystems 7300 Real-Time PCR system along with SYBR green PCR master mix. The threshold cycle number (Ct) was normalized to nuclear pore protein, *lamin2β receptor* (accession: KJ868807) (supplementary fig. S3B, Supplementary Material online) for endogenous gene expression, or *mCherry* mRNA for reporter gene expression (fig. 4*C*–*E*). Primer sequences are provided in supplementary table S2, Supplementary Material online.

### Immunofluorescence

*Patiria miniata* embryos were fixed in 4% paraformaldehyde/PBS for 20 min at RT, followed by permeabilization in 1% Triton X-100/PBS for 10 min. Embryos were then washed four times in PBS/0.1% Triton X-100 and post-fixed in ice cold methanol for 20 min. After another four washes, embryos were blocked in 3% BSA/PBS for 30 min and incubated with anti-*Pm*Tbr (1:500) overnight at 4 °C. Affinity purified polyclonal anti-*Pm*Tbr was produced in rabbits by Piece Custom Antibody Services. Embryos were washed four times and incubated in 1:100 FITC anti-rabbit (Sigma) overnight. Embryos were incubated in 1:10,000 DAPI (Life Technologies) for 30 min, washed four times in PBS/0.1% Triton X-100.

Embryos were imaged in Slowfade mounting media (Life Technologies) by confocal microscopy.

### Chromatin Immunoprecipitation PCR

ChIP was carried out as described by Mortazavi et al. (2006), with several modifications for sea star embryo samples. Chromatin extraction was performed as follows. Roughly 10^5 ^*P. miniata* embryos (∼10^8^ cells) were collected at 30 h postfertilization. These were cross-linked in 1% formaldehyde in artificial sea water for 10 min, stopped with 0.125 M glycine, collected by centrifugation, and washed 3× in cold PBS. Embryos were resuspended in lysis buffer (5 mM 1,4-piperazine-bis-[ethanesulphonic acid] [pH 8.0], 85 mM KCl, 0.5% NP-40, complete protease inhibitors [Roche Diagnostics]). After 10 min of lysis on ice, the embryos were passed through a 25-gauge needle 5–10 times and centrifuged to collect the crude nuclear preparation. Chromatin was digested to 500–100 bp pieces by micrococcal nuclease (New England Biolabs, Ipswich, MA) according to the SimpleChIP Enzymatic Chromatin IP Kit protocol (Cell Signaling Technology, Danvers, MA). The nuclear pellet was collected by centrifugation and lysed on ice for 10 min in 50 mM Tris (pH 8), 10 mM EDTA, 1% SDS (w/vol), and protease inhibitors. After the lysate was clarified by centrifugation, small aliquots were flash-frozen for immunoprecipitation, which was performed as described (Mortazavi et al. 2006).

Enrichment of the *PmOtxG* regulatory region was examined by PCR. A primer set was designed for an amplicon within the 850 bp CRM. Amplicons corresponding to regions 1 kb upstream and 1 kb downstream of *OtxG* were used as negative controls. Primer sequences are available in supplementary table S2, Supplementary Material online. PCR was performed for 30 cycles to achieve a linear range with the following conditions: 94 °C for 30 s, 58 °C for 30 s, and 72 °C for 20 s. All reactions contained 1 ng template (total chromatin, mock ChIP, or Tbr ChIP). Products were analyzed by 1% agarose gel.

## Supplementary Material

Supplementary tables S1 and S2 and figures S1–S3 are available at *Molecular Biology and Evolution* online (http://www.mbe.oxfordjournals.org/).

Supplementary Data
