# Supplementary material for: Modular Evolution of DNA-Binding Preference of a Tbrain Transcription Factor Provides a Mechanism for Modifying Gene Regulatory Networks
Source: Mol Biol Evol. 2014 Jul 12;31(10):2672–88. doi: 10.1093/molbev/msu213 (PMC4166925; doi:10.1093/molbev/msu213)
Supplement: Supplementary Data [file supp_31_10_2672__index.html]

Modular Evolution of DNA Binding Preference of a Tbrain Transcription Factor Provides a Mechanism for Modifying Gene Regulatory Networks. — Modular Evolution of DNA-Binding Preference of a Tbrain Transcription Factor Provides a Mechanism for Modifying Gene Regulatory Networks — Modular Evolution of DNA-Binding Preference of a Tbrain Transcription Factor Provides a Mechanism for Modifying Gene Regulatory Networks — Supplementary Data 

# Modular Evolution of DNA-Binding Preference of a Tbrain Transcription Factor Provides a Mechanism for Modifying Gene Regulatory Networks

## Supplementary Data

files

**Files in this Data Supplement:**

- Supplementary Data - pdf file
- Supplementary Data - pdf file
- Supplementary Data - pdf file
- Supplementary Data - docx file
- Supplementary Data - txt file
- Supplementary Data - xls file
