# Supplementary material for: Modular Evolution of DNA-Binding Preference of a Tbrain Transcription Factor Provides a Mechanism for Modifying Gene Regulatory Networks
Source: Mol Biol Evol. 2014 Jul 12;31(10):2672–88. doi: 10.1093/molbev/msu213 (PMC4166925; doi:10.1093/molbev/msu213)
Supplement: Supplementary Data [file supp_msu213_Sup_Figure_2_2.pdf]

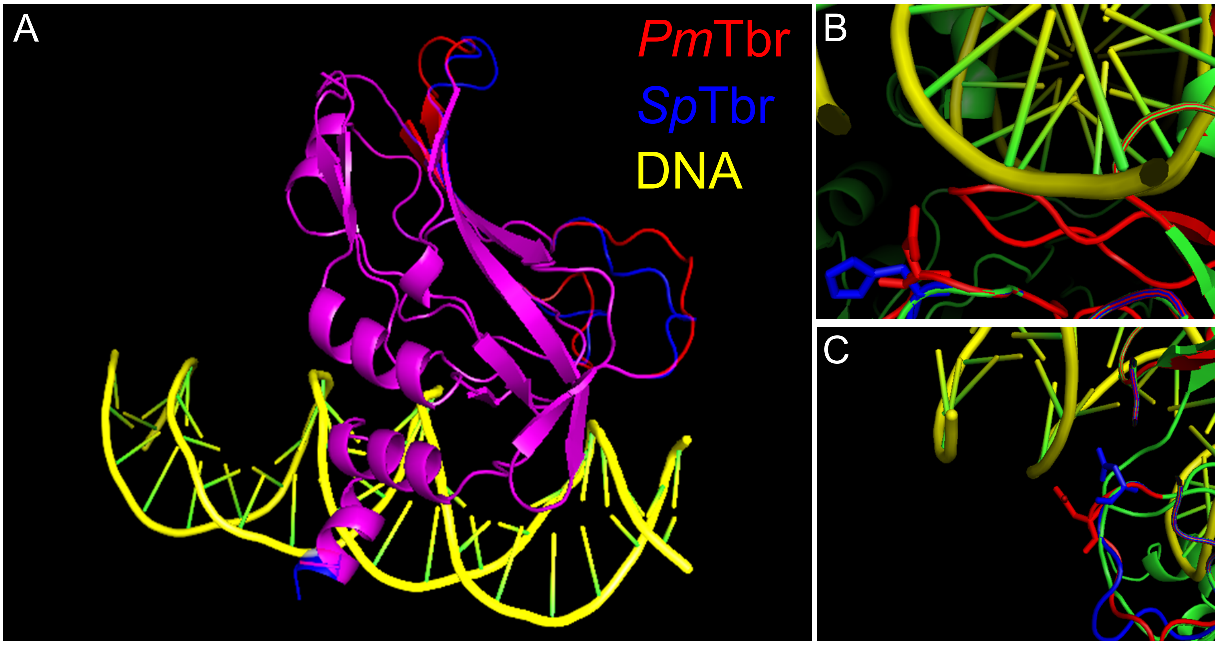

Supplemental Figure 2: Tbr Structural Prediction. A. Structure of sea star Tbr (red) and sea urchin Tbr's (blue) T-box domains modeled based on the structure of *Xl*Bra (PDB ID 1XBR)(Müller & Herrmann 1997) using Phyre (Kelley and Sternberg 2009). B. View of sea star asparagine 389 (red) vs. sea urchin histidine 479 (blue). The sea urchin amino acid is predicted to be poorly positioned to make a hydrogen bond with the DNA backbone. C. The adjacent amino acids (388/478) are also affected by this difference even though both orthologs have an asparagine in this position.
