# Supplementary material for: Modular Evolution of DNA-Binding Preference of a Tbrain Transcription Factor Provides a Mechanism for Modifying Gene Regulatory Networks
Source: Mol Biol Evol. 2014 Jul 12;31(10):2672–88. doi: 10.1093/molbev/msu213 (PMC4166925; doi:10.1093/molbev/msu213)
Supplement: Supplementary Data [file supp_msu213_Sup_Figure_3_2.pdf]

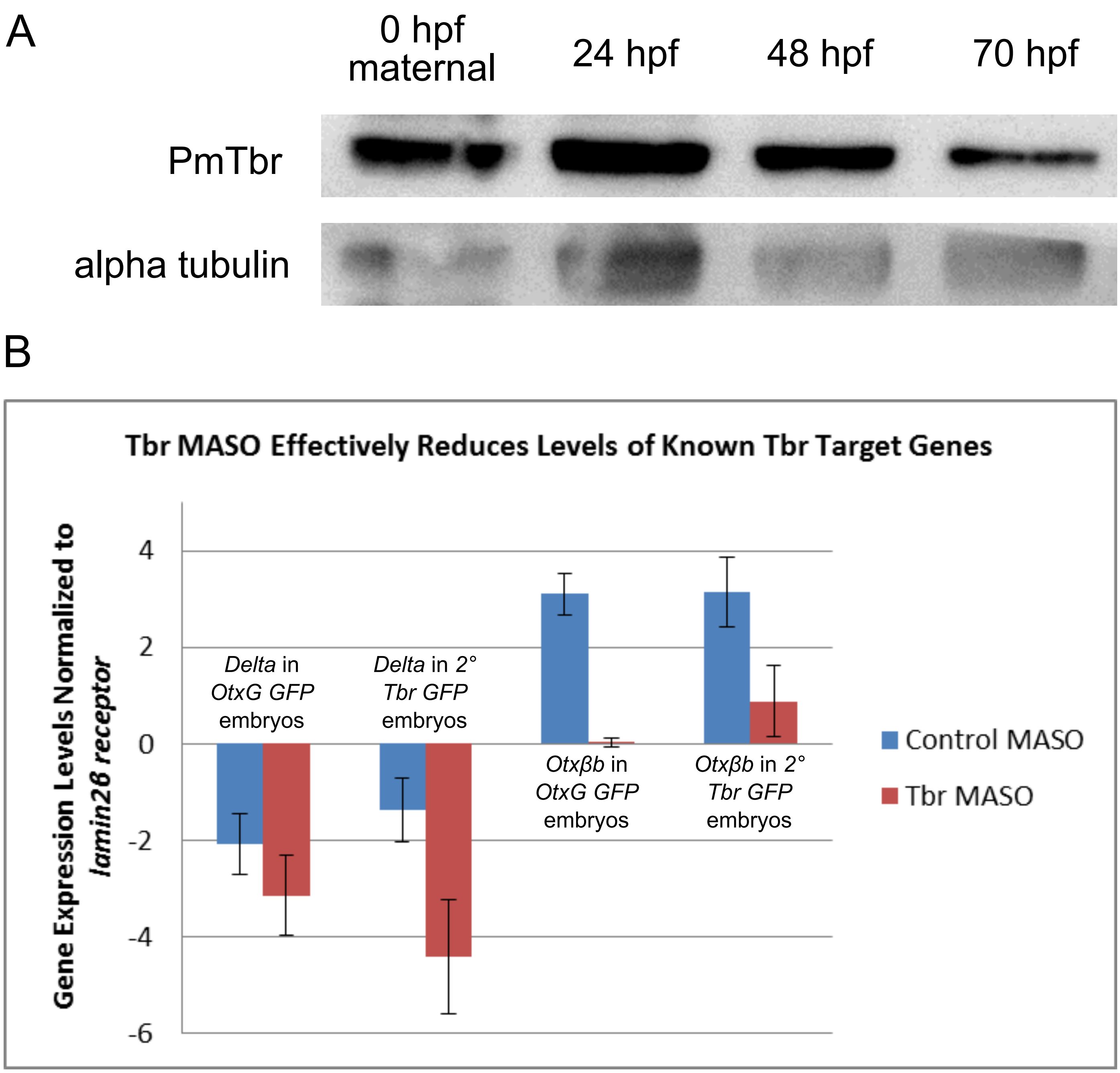

Supplemental Figure 3: A. Developmental Western Blot of *Pm*Tbr. Tbr levels are high maternally (0 hours post-fertilization (h)) and remain high through the gastrula stage (48 h). Levels are reduced at 70 h. Alpha-tubulin levels are shown as a loading control. B. Difference in Cycle Number Thresholds (Cts) of known Tbr target genes, *PmDelta* (Accession: ACC62396.1) and *PmOtxβb* (Accession: AY263968), normalized to *PmLamin2β receptor* (a nuclear envelope protein) (Accession: KJ868807). Levels are compared between control and Tbr morpholino oligonucleotide injected siblings. These same samples used to generate the *GFP* comparison in Figure 4E. Data points represent average of three experiments. Error bars indicate Standard Error of the Mean.
