# Supplementary material for: Modular Evolution of DNA-Binding Preference of a Tbrain Transcription Factor Provides a Mechanism for Modifying Gene Regulatory Networks
Source: Mol Biol Evol. 2014 Jul 12;31(10):2672–88. doi: 10.1093/molbev/msu213 (PMC4166925; doi:10.1093/molbev/msu213)
Supplement: Supplementary Data [file supp_msu213_Sup_Table_2_2.pdf]

Amplicon	Method	Forward Primer	Reverse Primer
GFP	qPCR	GCTGGCCGACCATTATCAACA	TGATCCCAGCAGCGGTCA
mCherry	qPCR	CCTCCTCCGAGCGGATGTAC	CGGGCTTCTTGGCCTTGTAG
PmLamin2β receptor	qPCR	GAGCATGCCTAAGCCAGACC	CTCCACCATGGGCTCCAGTA
PmDelta	qPCR	GTCAGGGTTCACTGGCATGT	CAGACATTGGTGGCCATCTT
PmOtxβb	qPCR	GAAAGGATGGATTGCGTCAT	ACCACTCATACTGCGGATTG
PmOtxG CRM set 1	ChIP RT-PCR	GCATAACCCTGCTTTCTGTTGCA	CCTGGCCTCCCATGTCCAATTG
PmOtxG CRM set 2	ChIP RT-PCR	CATGTGCAGCTTATCACTCGTCTG	AGAGGTGGTATTGGGATCTTGTCTG
PmOtxG 1kb upstream	ChIP RT-PCR	CGTGGCAGGCTCAGCAAGTG	GATGATGGCCCAAGCAATGTCATG
PmOtxG 1kb downstream	ChIP RT-PCR	CTCAAAATGGCGGGACGGTTACC	CTGCATGGTATGGCAGTGGAAT
2° Tbr GFP	Construct generation	GCCAGGGATGTCACCTCCTTGCTCGC	GCGAGCAAGGAGGTGACATCCCTGGC
Tbr Deletion	Construct generation	GCCAGGGATACCCACCCCTTGCTCGC	GCGAGCAAGGGGTGGGTATCCCTGGC

Supplemental Table 2: Primer Sequences
