# Supplementary material for: Modular Evolution of DNA-Binding Preference of a Tbrain Transcription Factor Provides a Mechanism for Modifying Gene Regulatory Networks
Source: Mol Biol Evol. 2014 Jul 12;31(10):2672–88. doi: 10.1093/molbev/msu213 (PMC4166925; doi:10.1093/molbev/msu213)
Supplement: Supplementary Data [file supp_msu213_Supplemental_Figure_legends.docx]

**Supplemental Figure 1: T-box Alignment used in Phylogenetic Tree Construction.** Alignment of DBDs from T-boxes of *Tbr*, *Bra*, and other T-box paralogs from a variety of deuterostome species. This alignment was used to construct the tree in Figure 1. All accession numbers are listed next to the gene name. Species abbreviations are as follows: *Bf*- *Branchiostoma floridae*, *Dr*-*Danio rerio*, *Hp*- *Hemicentrotus pulcherrimus*, *Lv*- *Lytechinus variegatus*, *Mm*-*Mus musculus*, *Pf*-*Ptychodera flava*, *Pj*- *Peronella japonica*, *Pl*- *Paracentrotus lividus,* *Pm*- *Patiria miniata*, *Pp*- *Patiria pectinifera*, *Sk*-*Saccoglossus kowalevskii*, *Sm*- *Scaphechinus mirabilis*, *Sp*-*Strongylocentrotus purpuratus*, *Xl*-*Xenopus laevis*, *Xt*- *Xenopus tropicalis*

**Supplemental Figure 2: Tbr Structural Prediction.** A. Structure of sea star Tbr (red) and sea urchin Tbr’s (blue) T-box domains modeled based on the structure of *Xl*Bra (PDB ID 1XBR)(Müller & Herrmann 1997) using Phyre (Kelley and Sternberg 2009). B. View of sea star asparagine 389 (red) vs. sea urchin histidine 479 (blue). The sea urchin amino acid is predicted to be poorly positioned to make a hydrogen bond with the DNA backbone. C. The adjacent amino acids (388/478) are also affected by this difference even though both orthologs have an asparagine in this position.

**Supplemental Figure 3:** A. Developmental Western Blot of *Pm*Tbr. Tbr levels are high maternally (0 hours post-fertilization (h)) and remain high through the gastrula stage (48 h). Levels are reduced at 70 h. Alpha-tubulin levels are shown as a loading control. B. Difference in Cycle Number Thresholds (Cts) of known Tbr target genes, *PmDelta* (Accession: ACC62396.1) and *PmOtxβb* (Accession: AY263968), normalized to *PmLamin2β receptor* (a nuclear envelope protein) (Accession: KJ868807). Levels are compared between control and Tbr morpholino oligonucleotide injected siblings. These same samples used to generate the *GFP* comparison in Figure 4E. Data points represent average of three experiments. Error bars indicate Standard Error of the Mean.

**Supplemental Table 1:** **All Ungapped 8-mer PBM Datasets for *Pm*Tbr and *Sp*Tbr and PWMs.**

**Supplemental Table 2: Primer Sequences**
